# How can Cytokine-induced killer cells overcome CAR-T cell limits

**DOI:** 10.3389/fimmu.2023.1229540

**Published:** 2023-08-22

**Authors:** Elisa Cappuzzello, Emilia Vigolo, Giulia D’Accardio, Giuseppe Astori, Antonio Rosato, Roberta Sommaggio

**Affiliations:** ^1^ Immunology and Molecular Oncology Unit, Veneto Institute of Oncology IOV - IRCCS, Padova, Italy; ^2^ Department of Surgery, Oncology and Gastroenterology, University of Padova, Padova, Italy; ^3^ Advanced Cellular Therapy Laboratory, Department of Hematology, San Bortolo Hospital of Vicenza, Vicenza, Italy

**Keywords:** CIK (cytokine-induced killer) cells, CAR (chimeric antigen receptor) T cells, ATMP, adoptive cell immunotherapy, hematological malignancies, solid tumors, GvHD, GMP

## Abstract

The successful treatment of patients affected by B-cell malignancies with Chimeric Antigen Receptor (CAR)-T cells represented a breakthrough in the field of adoptive cell therapy (ACT). However, CAR-T therapy is not an option for every patient, and several needs remain unmet. In particular, the production of CAR-T cells is expensive, labor-intensive and logistically challenging; additionally, the toxicities deriving from CAR-T cells infusion, such as cytokine release syndrome (CRS) and immune effector cell-associated neurotoxicity syndrome (ICANS), have been documented extensively. Alternative cellular therapy products such as Cytokine-induced killer (CIK) cells have the potential to overcome some of these obstacles. CIK cells are a heterogeneous population of polyclonal CD3^+^CD56^+^ T cells with phenotypic and functional properties of NK cells. CIK cell cytotoxicity is exerted in a major histocompatibility complex (MHC)-unrestricted manner through the engagement of natural killer group 2 member D (NKG2D) molecules, against a wide range of hematological and solid tumors without the need for prior antigen exposure or priming. The foremost potential of CIK cells lies in the very limited ability to induce graft-versus-host disease (GvHD) reactions in the allogeneic setting. CIK cells are produced with a simple and extremely efficient expansion protocol, which leads to a massive expansion of effector cells and requires a lower financial commitment compared to CAR-T cells. Indeed, CAR-T manufacturing involves the engineering with expensive GMP-grade viral vectors in centralized manufacturing facilities, whereas CIK cell production is successfully performed in local academic GMP facilities, and CIK cell treatment is now licensed in many countries. Moreover, the toxicities observed for CAR-T cells are not present in CIK cell-treated patients, thus further reducing the costs associated with hospitalization and post-infusion monitoring of patients, and ultimately encouraging the delivery of cell therapies in the outpatient setting. This review aims to give an overview of the limitations of CAR-T cell therapy and outline how the use of CIK cells could overcome such drawbacks thanks to their unique features. We highlight the undeniable advantages of using CIK cells as a therapeutic product, underlying the opportunity for further research on the topic.

## 1 Introduction

The last two decades have seen an unprecedented rise of novel therapeutic approaches to treat cancer due to the growing understanding of cancer immunology and immunotherapy. These results contributed to turning into reality the paradigm that a patient’s immune cells may represent effective “living drugs” against cancer cells. Nowadays, the most well-known immunotherapeutic approaches are represented by immune checkpoint blockade, adoptive cellular therapies (ACT), and cancer vaccines (1–3).

In the field of ACT, the successful treatment of patients affected by B-cell malignancies represented a breakthrough event that marked the beginning of a new era of therapeutic products, the Chimeric Antigen Receptor (CAR)-T cells (4). CAR-T cell therapies had an impressive development during the last decade, with an increasing number of clinical trials on different types of cancers (both hematologic and solid), diverse target antigens, and innovative genetic engineering approaches (5–8).

The remarkable results obtained with CD19 CAR-T cells led, in 2017, to the accelerated approval by the U.S. Food and Drug Administration (FDA) and the European Medicinal Agency (EMA) of two CD19-CAR-T cell drug products, tisagenlecleucel (tisa-cel, Kymriah) and axicabtagene ciloleucel (axi-cel, Yescarta). The relative approval studies (the ZUMA-1 trial for axi-cel (9) and the JULIET trial for tisa-cel (10)) reported complete responses (CR) up to 70-80% and significant improvement in overall survival (OS) in both adult and pediatric patients with disease relapse or refractory to other therapeutic interventions. However, the toxicity profile could be associated with cytokine release syndrome (CRS) and immune effector cell-associated neurotoxicity syndrome (ICANS). To date, six CAR T-cell therapies have been approved by the FDA, four of them targeting CD19-positive B-cell leukemias and lymphomas, and two targeting the B-cell maturation antigen (BCMA) expressed by multiple myeloma (11).

Despite the outstanding positive therapeutic improvements that have been achieved with the introduction of CAR-T cell therapeutic products into the clinical landscapes (12), CAR-T therapy is not an option for every patient, and several needs remain to be addressed. The CAR-T production process is expensive and labor-intensive (13), and a number of limitations have emerged from a clinical point of view (11, 14). T-cell expansion can be difficult when patients have been severely pre-treated, and therefore this became an exclusion criterion for patients who might be otherwise eligible (15). The severe toxicity resulting from CAR-T cell infusion has been documented extensively, and numerous studies focus on how to decrease the incidence and magnitude of CRS- and ICANS-related toxicity, and to better define the causes of such severe symptoms at a molecular and mechanistic level (16–19). Finally, the shift from an autologous to an allogeneic setting bring its own challenges, which have yet to be overcome (20). Alternative cell therapeutic products to CAR-T cells have the great potential to overcome some of these obstacles. Among them, cytokine-induced killer (CIK) cells proved to be very promising.

**Figure 1:**
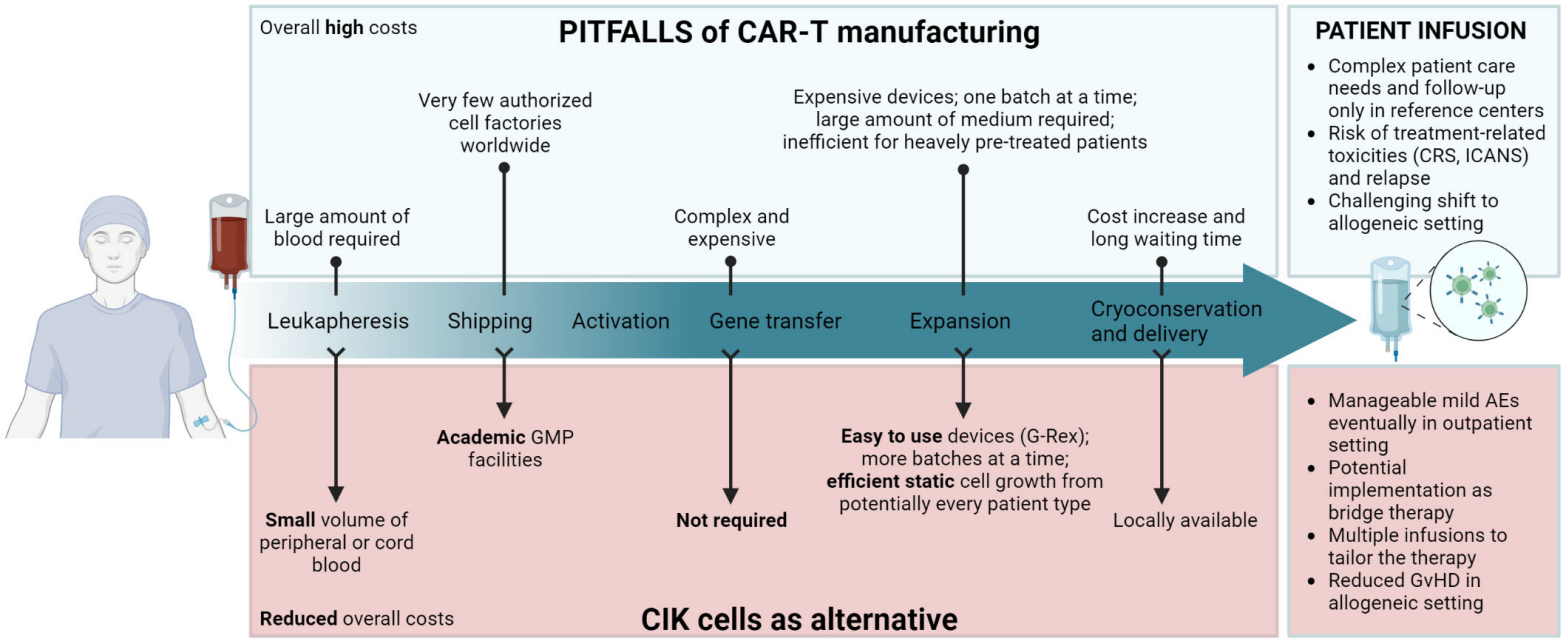
Schematic layout of CAR-T cell therapy’s main limitations during each step of manufacturing and infusion in patients (*upper panel*), and how the use of CIK cells could overcome such pitfalls (*lower panel*). Created with BioRender.com.

This review aims to give an overview of the limitations of CAR-T cell therapy and outline how the use of CIK cells could overcome such pitfalls thanks to their unique features, as depicted in **Figure 1**. We highlight the undeniable advantages of using CIK cells as a therapeutic product, underlying the opportunity for further research on the topic.

Importantly, we do not envisage a replacement of CAR-T cell therapy by the adoption of the CIK cell approach, as the clinical achievements of CAR-T cells are unquestionable and represented a critical breakthrough in the field. We rather encourage a side-by-side implementation of the CIK cell platform and a wider spread of this therapy, describing how CIK cells can fit in the landscape of ACT products, and how they could represent a realistic alternative option for those patients who cannot access CAR-T cell therapy, as well as to function as bridge therapy for allogeneic transplantation or consolidation after CAR-T cell therapy.

## 2 Defining CIK cells

In 1991, Schmidt-Wolf and Negrin reported the optimization of the protocol of the lymphokine-activated killer (LAK) cells, which led, for the first time, to the expansion of CIK cells. They demonstrated that CIK cells show an enhanced proliferation capacity and increased cytotoxic activity both *in vitro* and *in vivo* when compared to former LAK cells (21).

CIK cells are a heterogeneous population of polyclonal CD3^+^CD56^+^ T cells with phenotypic and functional properties of NK cells, which can be expanded from different sources including peripheral blood, bone marrow, and cord blood, through a precise expansion protocol involving the timed addition of interferon-γ (INF-γ), anti-CD3 antibodies and interleukine-2 (IL-2) (22–27). Following 14 days of culture, the bulk CIK cell population is mainly composed of CD3^+^CD56^+^ CIK cells, CD3^+^CD56^-^ T cells, and a small fraction of CD3^-^CD56^+^ NK cells. It has been demonstrated that CD3^+^CD56^+^ CIK cells derive from CD3^+^ T cell precursors, which acquire the CD56 expression during expansion (25, 28), even though a clear molecular signature has not yet been described.

### 2.1 CIK cell cytotoxicity

The great peculiarity of such effector cells is the major histocompatibility complex (MHC)-unrestricted lytic capacity towards a wide range of hematological (21, 25, 29–33) and solid (27, 34–38) tumor cells, without the need of prior antigen exposure or priming. CIK cell cytotoxic capacity is exerted by the engagement of natural killer group 2 member D (NKG2D) molecules, which recognize specific ligands known as UL16-binding protein family members (ULBP1–6) and MHC class I-related molecules A and B (MIC A/B) widely expressed on tumor cells (39–42). To better define the functional characteristic of the different cell fractions present in bulk cultures, in a preclinical study the CD3^+^CD56^+^ and CD3^+^CD56^-^ subpopulations were sorted, and their cytotoxicity tested both *in vitro* and *in vivo* against hematological malignancies, to be compared with unsorted bulk CIK cells (35). Results showed that the CD3^+^CD56^+^ cells retain the killing activity due to the high expression of NKG2D, and are endowed with low proliferative capacity. The CD3^+^CD56^-^ subpopulation instead showed a low anti-tumor capacity while expressing NKG2D, suggesting that the latter cannot be the only player accounted for CIK cytotoxicity (35). Moreover, this population is highly proliferative, since even the smallest amount of CD3^+^CD56^-^ cells that remained in culture after cell selection were able to proliferate in the tumor of the injected mice (32). Nevertheless, studies have shown that each subpopulation is required for proper differentiation, proliferation, and ultimately tumor killing, emphasizing the benefit of infusing the bulk CIK cell culture rather than sorted subpopulations (28, 43). Similar conclusions were drawn in the context of solid tumors, such as colorectal cancer, by Sangiolo and colleagues (34, 35), underlying the different roles of the distinct CIK cell subpopulations.

In addition to NKG2D, other NK-specific receptors including NKp30 and DNAM-1 are expressed, albeit at lower levels when compared to NK cells (28). The co-stimulatory molecule DAP10 and the ICAM-1 ligand CD244 (2B4) could also in part be responsible for the CIK cytotoxicity (39, 42), but more detailed studies are needed. Indeed, Wu et al. showed that 2B4 alone is not able to stimulate CIK cell cytolytic activity but can synergize with NKG2D in certain circumstances, and, in turn, induce LFA-1 expression on CIK effectors (39). The crucial role of LFA-1 in CIK cells for functional binding and cytotoxicity is clear, but it has been hypothesized that also other surface molecules are involved in binding, since its surface expression does not correlate with the cytotoxic activity (44).

Strikingly, CIK cells express T-cell receptor (TCR) in a polyclonal fashion and with a similar proportion of αβ and γδ chains as in peripheral blood T cells (28, 44). Nevertheless, a seminal work from Negrin’s lab showed that the inhibition of TCR downstream activation does not affect CIK cell cytotoxicity, demonstrating that their lytic activity mainly relies on a non-MHC restricted killing mechanism, rather than a TCR/MHC-restricted modality (44, 45). Finally, CIK cell activation and engagement with the target cell leads to the release of granzymes and perforins that provoke cell lysis (45).

Moreover, it has been reported that CIK cells express the FcγRIIIa (CD16a) receptor. Cappuzzello et al. showed that the CD16a expression is highly donor-dependent, and remains stable during the entire period of the expansion protocol (27, 33, 36). The CD16a receptor can be easily engaged by monoclonal antibodies (mAb), becoming, therefore, a powerful mediator for triggering a potent antibody-dependent cell-mediated cytotoxicity (ADCC) against tumor cells (27). Thus, using mAbs and other more recently developed immunotools, CIK cells can be readily redirected toward diverse tumor types and their cytotoxic capacity can be further amplified (27, 33, 36, 46). These combination strategies involving CIK cells will be further described in this review.

### 2.2 Reduced induction of GvHD

The great potential of CIK cells when compared to other cellular therapy products lies in the very limited ability to induce graft-versus-host disease (GvHD) in the allogeneic setting (47). On one hand, the expression of chemokine receptors and adhesion molecules empower the CIK cells with strong homing capacity towards the targeted tumor tissue, lymph nodes, and spleen. On the other hand, the homing and infiltration into healthy organs is minor, therefore limiting a GvHD reaction. Nishimura et al. demonstrated in Balb/c mice that both splenocytes and CIK cells are able to traffic to the GvHD target organs such as the liver, spleen and gastrointestinal tract, but CIK cells showed a lower proliferation rate, a higher number of early apoptotic events, and a higher and more sustained IFN-γ production when compared to splenocytes (47). Indeed, the IFN-γ produced by CIK cells is crucial in the protection from GvHD as CIK cells obtained from IFN-γ knock-out mice cause a lethal acute GvHD (aGvHD) (48–50).

Furthermore, it has been shown that CIK cells exhibit very low alloreactivity across human leukocyte antigen (HLA) barriers when compared to conventional donor lymphocyte infusion (DLI) (35, 51), as confirmed by preclinical and phase I/II studies where the infusion of bulk CIK cells population was well-tolerated (43, 52–56).

Altogether, a deeper insight into the molecular patterns involved in CIK cell activity not only during expansion but also during interaction with target cells, would improve our understanding of their behavior and provide opportunities to enhance their immunotherapeutic potential.

## 3 Manufacturing perspective

### 3.1 Regulatory framework and logistics

The EMA defines as “Advanced Therapy Medicinal Products” (ATMPs) any drug for human use, which are based on genes, tissues, or cells. The European Union (EU) Regulation 1394/2007 defines the guidelines for their centralized marketing authorization, supervision and pharmacovigilance, and establishes that only accredited and authorized facilities can manipulate tissues or cell therapies according to Good Manufacturing Practice (GMP) (57, 58). In particular, CAR-T cells are classified as gene therapy medicinal products and CIK cells as a cellular therapy medicinal product. The understanding of the distinctive features of CIK cells and the experience acquired with this cell therapy in clinical trials underline the extremely compelling potential of this effector cell population, placing them as an alternative therapeutic approach to CAR-T cells in specific clinical settings (**Figure 1**).

It is well known that the manufacturing process of autologous CAR-T cells presents clinical and logistical hurdles. For the latter, the current logistic organization follows a centralized manufacturing model where the starting material, namely the patient’s cells, is collected by leukapheresis at the hospital and shipped frozen to the manufacturing site. Once produced, the ATMP is shipped back cryopreserved to the clinic and administered to the patients (11, 59). Only a few manufacturing sites are authorized for the CAR-T cell production, which results in a shortage of manufacturing slots and cumbersome logistics, leading to extremely high costs (11, 20). To move away from this centralized scheme, a point-of-care manufacturing model should be envisaged, so that the geographical proximity of the manufacturing site and of the point-of-care would limit the costs of the shipment across international borders of both the starting material and the ATMP (59). To this end, the use of an ATMP that can be conveniently manufactured in local GMP facilities would allow a more accessible therapy for patients.

Actually, CIK cells are produced in academic GMP facilities, authorized by the National competent authorities, and CIK cell treatment is now licensed in many countries (22, 59). Moreover, simple and extremely efficient expansion protocols have been recently developed, and they will be further described in this review (60, 61).

### 3.2 Selection and follow-up of patients

Besides the shortage of manufacturing slots, the accessibility to CAR-T cell therapy is restricted to patients who have a higher probability to achieve a clinical response and a lower risk of treatment-related toxicities or relapse. Often these are heavily pre-treated patients, and it is not always possible to generate clinically relevant doses of CAR-T cells. Thus, manufacturing failures of CAR-T cells have to be foreseen, since, in this case, alternative and bridge therapies need to be considered (62). Conversely, the expansion of CIK cells, even from heavily pre-treated patients, has been already described, reaching the clinically relevant doses with standard culturing protocols (22).

Patients are also evaluated for their risk of developing and tolerating therapy-related toxicities, which are one of the most challenging factors of CAR-T cell therapy. Upon infusion, CAR-T cells can undergo rapid *in vivo* proliferation that can be associated with severe and life-threatening adverse events, such as CRS, ICANS, prolonged cytopenia, and bacterial, fungal, and viral infections. CRS represents the most common side effect with a range of incidence of 50-90% among the different clinical studies (16, 17, 63–68). Thus, patients are generally admitted to the inpatient unit during the pre- and early post-infusion period to identify the onset of toxicities and manage them in the shortest time. Reference centers are required for the infusion of CAR-T cells to handle the complex scheduling logistics and patient care needs, which include the availability of an on-site hematology unit, an intensive care unit, neurological and emergency departments, a pharmacy, and blood transfusion center, in which all categories of personnel including scientists, nurses, and physicians have to be appropriately trained, thus limiting the number of hospitals that could safely administer such treatments (65, 69, 70). The scientific community is working to develop strategies to reduce toxicities and thus move toward an outpatient administration of cellular therapies, where infused patients do not require overnight hospitalization, reducing the time and costs associated with inpatient stays and patient monitoring (71). The related toxicities observed for CAR-T cells are not present in CIK cell-treated patients. Clinical trials occasionally reported the incidence of only minor side effects, which include mild hypotension, fever and chills, headaches, nausea, and vomiting that do not require intensive medical interventions (72). The use of an effector cell population with a safer profile, such as CIK cells, would thus foster the delivery of cell therapies in the outpatient setting.

### 3.3 Economic considerations

An additional limitation of CAR-T cell therapy is the pricing, since a single infusion is estimated at US$ 373,000 for axi-cel and US$ 475,000 for tisa-cel, with similar prices for other authorized products (14). Moreover, considering the supportive care required in most cases to manage the therapy-associated toxicity and the fact that many patients do not remain in remission after CAR-T cell therapy, such costs are certainly much higher (14). Indeed, approximately 30-50% of patients who achieve a complete response following tisa-cel or axi-cel do not achieve long-term remission and ultimately relapse (73, 74).

On the contrary, the expansion of CIK cells requires a drastically lower financial commitment, as the manufacturing does not involve the engineering with expensive GMP-grade viral vectors in centralized manufacturing facilities (75, 76). We estimated that the cost of consumables and reagents, which include GMP-grade culture medium, plasticware, cytokines, freezing media, and antibodies, is less than €5,000 per batch. This cost has to be integrated with the costs of the personnel, quality control, GMP facility--related costs, and plant depreciation, which can vary from one academic facility to another. Although it is not possible to thoroughly compare the price of an ATMP produced in academic cell factories, such as CIK cells, and an ATMP with a global market size, such as CAR-T cells, we hypothesize that the overall cost for the expansion of a batch of CIK cells is of an order of magnitude less than CAR-T cells.

Moreover, the manufacturing of CIK cells leads to a massive expansion of the effector cells, which allows multiple doses to be prepared and patients to be treated with multiple infusions rather than a single infusion, as it is the case with CAR-T cells. Even using multiple infusions of CIK cells, the dose-limiting toxicity was not reached in clinical trials, suggesting that higher doses may actually be used (54). The administration of several cycles of CIK cells could help tailor the therapy to the patient by adjusting the cell dose, improving the control of the disease in the long term, and consolidating the clinical response avoiding relapses and related costs.

### 3.4 Additional considerations on the starting material

For the expansion of CAR-T cells, it is necessary to perform a lymphapheresis, a procedure that takes hours, processes volumes ranging from 3 to 25 liters, and aims at collecting a minimum of 0.6x10^9^ and a target of 2x10^9^ CD3^+^ cells (77). The status of the patient, who is often heavily pre-treated and cytopenic, the quantity and the quality of CD3^+^ cells collected, and the purity of the apheresis, in particular the quantity of contaminating myeloid cells, are all factors that affect the successful transduction and expansion of CAR-T cells, potentially leading to manufacturing failures and thus the withdrawal of the patient from the treatment (59, 77, 78). On the contrary, apheresis is not necessary for the expansion of CIK cells, since as low as 30 ml of peripheral blood is sufficient to produce enough CIK cells to repeatedly treat a 70-kg patient when administered at a concentration of about 5x10^6^ cells/kg, as already performed in previous clinical trials (54, 60).

The first preclinical studies on human CIK cells were conducted starting from peripheral blood-circulating mononuclear cells (PBMCs) of healthy donors, subsequently, from PBMCs obtained from leukemia patients (26, 79), and later from heavily pre-treated patients (60). The use of peripheral blood represents an undoubtedly easier procedure to access the starting material, and extremely smaller volumes need to be manipulated compared to apheresis, still allowing to obtain clinically relevant doses of CIK cells (60). Details on the expansion protocols will be described in a dedicated section below.

To overcome the risk of blast contamination in the drug product, and to overcome the pitfalls related to the manufacturing of autologous products, scientists have been investigating allogeneic, “off-the-shelf” CAR-T cells from healthy donors, in order to allow large-scale production, reduce costs and time for the therapy availability, and finally achieve a higher level of standardization (11, 20). The main risk of allogeneic products is the development of GvHD, due to the HLA mismatch between donor and recipient. Additionally, there could be a risk of reduced persistence and therefore efficacy of the CAR-T cell therapy whether it occurs the rejection of the allogeneic CAR-T cells (11). Two main approaches have been evaluated for B-cell malignancies, namely the use of CAR-T cells modified by TCR α-chain knockout, or the use of alternative cell populations lacking allogeneic reactivity, such as NK or γδ T cells; both strategies, however, have their own manufacturing challenges, including prolonged culture times and relative resistance to transduction (57, 80). In terms of product manufacturing, CIK cells appear to have advantages over these alternative cell populations, since they do not require selection during or after expansion, as it occurs for CAR-NK or CAR-γδ T cells, and have a higher proliferation capacity that allows to easily reach clinical relevant doses (51). Moreover, it has been reported that at the end of the expansion period, malignant cells are not present in CIK cell cultures (26, 33).

As previously described in this review, one of the most distinctive features of CIK cells when compared to other cell therapy products, is the very limited ability to induce GvHD in an allogeneic setting (47), as confirmed by several clinical trials treating patients with HLA-matched, haploidentical or unmatched CIK cells (52–56). In the first published phase I study with allogeneic CIK cells, the infusion of donor-derived CIK cells demonstrated some clinical activity in half of the patients, with aGvHD that never exceeded grade 2 (52) and even lower incidences in subsequent studies (54, 55, 81). Moreover, CIK cells have been isolated and clinically tested also when derived from umbilical cord blood (UCB) (24, 82), which represent an optimal starting material for the expansions of cells for allogeneic immunotherapy due to the lower immunogenicity; thus, a higher degree of HLA-mismatch between the recipient and donor can be tolerated (53). UCB has also the potential to provide products with “off-the-shelf” availability by cryopreservation of *ex vivo* expanded UCB cells (83). Several studies demonstrated that CIK cells can be efficiently expanded from small volumes of freshly collected UCB (10-15 ml), or even from the washouts of bags used for UCB transplant. UCB-derived CIK cells showed a phenotype and an antitumor activity comparable to their peripheral blood counterparts (24). Starting from a very small percentage of total nucleated cells, it was possible to expand enough cells to treat patients with multiple infusions of CIK cells (53).

### 3.5 Additional considerations on the devices for cell expansion

The ATMP regulation requires GMP manufacturing of cells for immunotherapy. The expansion of millions or billions of cells, which is the number necessary to treat a patient, would require the use of roughly one hundred of conventional T175 flasks. To handle such a huge number of flasks is cumbersome and exhibits numerous limitations in the GMP setting. Thus, several closed systems have been developed that drastically reduce cell manipulation, thus limiting the risk of microbial contamination, as well as ensuring a higher level of standardization. Bioreactors, such as Xuri or Prodigy, rely on the mechanical rocking of culture vessels to guarantee adequate distribution of nutrients and gas exchange during cell expansion (84, 85). However, bioreactors are expensive, require large amounts of fresh medium, frequent cell density adjustments, and usually allow only one batch of cells to be expanded at a time; thus, GMP facilities need to have several bioreactors to guarantee the production of more than one batch. Additionally, bioreactors must be subjected to periodical qualification. Hence, alternative systems for GMP-compliant expansion of cells have been developed, such as Gas-permeable rapid expansion G-Rex devices (Wilson Wolf), which are disposable, GMP-compliant, quite simple closed-culture vessels that can be accommodated in standard incubators. They are provided with a flat, gas-permeable silicone membrane at the base of the vessel that allows an efficient gas exchange. The structure of the G-Rex flasks allows an increased depth of the medium above cells, thus optimizing cell proliferation and survival. Moreover, cells in G-Rex grow statically, favoring the proliferation of lymphocytes, which tend to grow in clusters (86). G-Rex culture devices have been used for the *ex vivo* expansion of many cell types, such as cytotoxic T cells (87, 88), tumor-infiltrating lymphocytes (89, 90), regulatory T cells (91), NK cells (92) and also CAR-T cells (93, 94).

Two reports have been published on the expansion of CIK cells in G-Rex devices. Palmerini et al. demonstrated for the first time the efficacy and feasibility of the expansion of CIK cells from a small volume of healthy donor peripheral blood using a serum-free protocol, and compared the results to the standard cultures in conventional T-flasks (60). The culture of CIK cells in G-Rex led to a significant more efficient cell expansion, with a 752-fold increase in cell number, an enrichment in CD3^+^CD56^+^ CIK cells, a higher proportion of CD8^+^ cells, and a less differentiated phenotype, which could contribute to long-lasting therapeutic responses and *in vivo* persistence. Notably, in the protocol described by Palmerini et al., differently from other protocols, CIK cells are efficiently expanded using a serum-free medium, which eliminates the risk of viral infection and the batch-to-batch variability, since the composition of supplements such as AB serum, frozen plasma or platelet lysate (95) is highly batch-dependent. The protocol described was upgraded to successfully expand in G-Rex CIK cells from small amounts of peripheral blood from B-cell malignancy patients with extremely low CD3^+^ counts and high tumor burden. Indeed, the early addition in the culture of Blinatumumab, a bispecific antibody that simultaneously targets CD3 on effectors and CD19 on target cells, led to the concomitant expansion of clinically relevant numbers of CIK cells, and the complete elimination of the malignant B cell fraction, without any magnetic selection or cell sorting at the beginning of the expansion (33).

Gotti et al. confirmed the feasibility of CIK cell expansion in G-Rex devices developing a protocol that further reduces culture manipulation, using lactate as an indicator of cell growth (61). CIK cells, which were expanded starting from both peripheral blood and cord blood, showed a similar phenotype for most activation markers, adhesion molecules, and checkpoint inhibitors, and a T cell subset composition similar to that of CIK cells cultured from flasks. Gotti and colleagues also demonstrated the therapeutic activity *in vivo* in an orthotopic model of pre-B acute leukemia, and confirmed the lack of GvHD in contrast to unmanipulated mononuclear cells (61).

## 4 CIK cell clinical trials

The first phase I clinical trial using CIK cells was conducted in Germany in 1999, by infusing CIK cells modified with the IL-2 gene for the treatment of 10 patients with metastatic renal carcinoma, colorectal cancer, and lymphoma. Clinical outcomes showed that six patients underwent progression, three patients showed no change by treatment, and one lymphoma patient developed a complete response. As stated by the authors, this result was promising for patients with chemotherapy-resistant and progressive metastatic disease. Except for three patients who developed grade 2 fever that spontaneously resolved the next day, no other adverse events (AE) were reported. This pioneering study paved the way for further investigations on the potential of CIK cell therapy (96). Indeed, the remarkable safe profile, as well as the therapeutic efficacy of CIK cells, has been extensively confirmed in many different clinical settings, highlighting the limited incidence of severe GvHD as the main advantage of this cellular therapy (82). The most relevant clinical trials, which are discussed in this review, are reported in **Table 1**.

**Table 1:** Clinical trials based on CIK cell immunotherapy.

Reference	Tumor type	CIK-treated patients (n)	Treatment schedule	Efficacy	Safety
Laport et al. (81)	relapsed allo-HSCT	18	dose-escalating, from 1x10^7^ CD3^+^ cells/kg to 1x10^8^ cells/kg	mOS: 28 months CR: 27.7%	aGVHD grade 1/2: 11% cGVHD: 5.5%
Narayan et al. (97) NCT01392989	Myeloid Neoplasms	44	one CIK cell infusion (12.4x10^8^/kg) after conditioning	2-year OS: 52.6%	aGVHD: 16.3%
Merker et al. (98)	relapsed allo-HSCT	36	CIK cells (16x10^6^/kg), median of 2 and maximum of 9 cycles	CR: 53%	aGVHD: 25%
Introna et al. (54)	relapsed allo-HSCT	73	sequential infusion of DLI (1x10^6^/kg) followed by dose-escalating CIK cells (1 to 5x10^6^/kg), for 3 cycles	CR: 26%, PR: 4%, stable disease: 11%. 1- and 3-year PFS: 31% and 29%. 1- and 3-year OS: 51% and 40%.	aGVHD: 16%
Wang et al. (56)	NSCLC	133 (auto) 170 (allo)	autologous or haploidentical, CIK cells 5x10^9^ cells/cycle, 4 cycles	mOS: auto 11 months, allo 8 months	mild AEs, no differences allo vs auto (P>0.05)
Lee et al. (99) NCT00699816	HCC	114	autologous CIK cells, 6.4x10^9^ cells/cycle,16 cycles in total	mDFS: 44 months	AEs grade 1 or 2: 47%
Chen et al. (100)	HCC	102	1.0 to 1.5x10^10^ CIK cells per cycle, at least 4 cycles, transfused after tumor resection	1-, 3-, and 5-year DFS: 85.3%, 68.2%, and 60.4%. 1-, 3-, and 5-year OS: 99.0%, 93.0%, and 84.3%.	mild and self-limiting AEs
Li et al. (101)	NPC	112	GC followed by at least 4 cycles of CIK cells	mPFS: 21 months mOS: 32 months	no acute or chronic infectious cases
Zhou et al. (102)	Epithelial Ovarian cancer	72	3-6 cycles of chemotherapy followed by sequential CIK cells (range 8.0×10^9^ -1.3×10^10^ cells/cycle, 4 cycles)	mOS, 63.6 months mPFS: 41.6 months	AE grade 1 and 2: 12.5%, no treatment-related AE
Wang et al. (103)	Advanced pancreatic cancer	25	CIK cells plus chemotherapy (gemcitabine and/or S-1)	mOS: 13.5 months DCR: 68.0%	mild AEs
Kong et al. (104) NCT00807027	GBM	91	standard chemoradiotherapy with TMZ plus 14 cycles of 10^9^~2x10^10^ cells	mOS: 22.5 months mPFS: 8.1 months DCR: 82.4%	AE ≥grade 3: 47.1%
Zhao et al. (105)	mRCC	29	anti-PD-1 treatment plus CIK cells, median 6.4×10^9^/cycle	CR: 24.1%, PR: 17.2% mPFS: 15 months mOS: 37 months	AEs grade 1 and 2: 86.2% AEs grade 3: 3.4%
Zhou et al. (106) NCT03987867	advanced NSCLC	34	anti-PD-1 treatment plus CIK cells ≥ 1×10^10^ total, and chemotherapy, 4 cycles	ORR: 82.4% DCR:100.0% mPFS:19.3 months	grade 3 or greater AEs
Lin et al. (107)	stage IV breast cancer	188	DC-CIK treatment, 3 cycles minimum	5-year DFS: 42% 5-year OS: 44%	mild AEs
Jiang et al. (108) NCT01781520	advanced pancreatic cancer	47	DC-CIK mean of 7.8x10^9^/cycle plus S-1 chemotherapy	6-month OS: 62.2% 6-months PFS: 41.6%	no grade 3 adverse effects
Li et al. (109)	CRC	3203	CIK, DC-CIK ± chemotherapy	improvement of OS, PFS, and ORR	AE rate: 53.5%
Magnani et al. (110) NCT03389035	B-ALL relapsed allo-HSCT	13	allogeneic CAR CIK-CD19, 4-dose escalation: 1×10^6^, 3×10^6^, 7.5×10^6^, and 15×10^6^/kg	ORR: 61.5%	AEs grade 1/2. No GVHD, neurotoxicity, or dose-limiting toxicities

CR, complete response; DCR, disease control rate; DFS, disease-free survival; GC, gemcitabine plus cisplatin chemotherapy; HCC, Hepatocellular Carcinoma; HSCT, hematopoietic stem cell transplant; mOS, median OS; mRCC, metastatic Renal Cell Carcinoma; NPC, Nasopharyngeal Carcinoma; NSCLC, Non-small cell Lung Cancer; ORR (CR+ PR), overall response rate; OS, overall survival; PFS, progression-free survival; PR, partial response; TMZ, temozolomide.

In 2010, the International Registry on CIK Cells (IRCC, www.cik-info.org) was established to collect exhaustive information on CIK cells clinical trials and to attempt standardization of CIK cell-based treatments (72, 75, 111). The 2020 report (72) summarized the results of 106 clinical trials enrolling a total of 10,225 patients, of which 4,889 (47.8%) were treated with CIK cell therapy alone or in combination with other conventional or novel therapeutic strategies. In these trials more than 30 kinds of cancers were included (72). A significant improvement in median progression-free survival (mPFS) and median overall survival (mOS) were reported in the majority of the studies. Besides this, 10 studies reported a significantly increased 1‐year survival rate, and 9 studies reported a significantly increased 5‐year survival rate, highlighting the therapeutic efficacy of CIK cell therapy (72).

### 4.1 CIK cells in relapsed allo-HSCT patients

Allogeneic-hematopoietic stem cell transplantation (allo-HSCT) represents a curative treatment for patients with hematologic malignancies, but relapse remains one of the leading causes of treatment failure. Strategies to induce remission after relapse include DLI, which however often induces aGvHD and, in turn, treatment failure. A phase I dose-escalating trial showed how CIK cells could be safely administered in the relapsed allo-HSCT setting, being associated with a low incidence of aGvHD. Indeed, in a cohort of 18 patients, aGvHD of grade 1 or 2 was observed in two patients, and five patients achieved or maintained a CR for more than one year after CIK infusion (81). Furthermore, in 2019, the same group proposed CIK cells as early post-transplant consolidation therapy. They showed that this treatment regimen is well tolerated, has antitumor activity, and promotes early donor chimerism without significantly affecting the rates of aGvHD in a cohort of 44 patients (NCT01392989) (97). Indeed, CIK cell therapy showed similar, or even improved, graft-versus-leukemia (GVL) effects when compared to DLI treatment in patients with a low tumor burden, observing a significantly reduced 6-month cumulative incidence of relapse (CIR) following allogenic HSCT (98).

Interestingly, Introna et al. in a phase IIA study investigated the therapeutic potential of the sequential infusion of a small amount of DLI followed by CIK cells in relapsing allo-HSCT patients. The authors demonstrated that this combinatorial approach is safe, and shows significant efficacy in patients with low tumor burden (CR: 26%) with a remarkably low severe acute and chronic GvHD incidence. In most cases, aGvHD developed after DLI infusion, and subsequent administration of CIK cells did not induce further aGvHD. In this regard, they also found that haploidentical CIK cells did not cause aGvHD even at higher doses, highlighting a significant difference compared to standard DLI treatment (54).

### 4.2 CIK cells in solid tumor patients

CIK cells have demonstrated their safety and efficacy in several clinical trials where patients with solid tumors were enrolled. According to the IRCC reports, the tumor entities investigated in most CIK cell studies are lung cancer (26.4%), hepatocellular carcinoma (HCC, 18.9%), renal cell carcinoma (RCC, 15.1%) and lymphoma (15%) (72, 75, 111).

In the study from Wang et al., non-small cell Lung Cancer (NSCLC) patients were treated at different stages with either autologous or allogeneic CIK cells, observing no significant differences in the AE between treated and control groups, with a mOS of 11 months and 8 months, respectively (56).

Moreover, in a multicenter, randomized, open-label, phase III trial with 114 HCC patients (NCT00699816), Lee et al. showed that CIK cell treatment induced a 14-months median disease-free survival (mDFS) improvement compared to control group, which did not receive any CIK cells infusions (44 months vs 30 months). The CIK cell immunotherapy reduced all types of tumor recurrence and, moreover, the reported AEs were mild to moderate (grade 1 or 2) (99).

The efficacy of CIK cells for the treatment of HCC was confirmed also by Chen et al. in a retrospective study on 102 patients who received CIK treatment after curative resection, observing a significant improvement in survival. Specifically, the 1-, 3-, and 5-year disease-free survival (DFS) rates were 85.3%, 68.2%, and 60.4% respectively, with a marginal significant difference between CIK and control group (P=0.055). Also, the CIK group exhibited significantly higher OS than the control group, underlying the efficacy of CIK cell therapy (100).

### 4.3 CIK cell combination therapies

The low toxicity and high feasibility of CIK cell treatment have encouraged the study of novel combination therapies, which are showing higher efficacy compared to monotherapy. The effects of the combination of CIK cells and chemotherapy on metastatic Nasopharyngeal Carcinoma (NPC) was investigated in a retrospective study with 222 patients, among whom 112 received gemcitabine plus cisplatin (GC) regimen chemotherapy followed by at least 4 cycles of CIK immunotherapy. Over the 3-year follow-up period, the GC plus CIK therapy group had significantly higher survival rates than the GC alone group (mPFS 21 months vs 15 months; mOS 32 months vs 23 months), which indicated that CIK adjuvant immunotherapy could effectively maintain disease stability and prolonged survival in advanced metastatic NPC patients (101).

Similarly, ovarian cancer patients treated with CIK cells combined with a chemotherapeutic regimen exhibited a significantly more favorable OS and PFS than control group who received only chemotherapy (mOS, 63.6 vs 39.6 months and mPFS, 41.6 vs 26.1 months) (102).

In addition, a study conducted by Wang et al. revealed a significant improvement in disease-control rate (DCR) in advanced pancreatic cancer patients (68.0%) treated with a combination of CIK cells and gemcitabine and/or the oral chemotherapy S-1, compared with the control chemotherapy group (29.8%) (103).

In a multi‐center, Phase III trial on a total of 180 patients with newly diagnosed glioblastoma, autologous CIK cells infusion combined with standard radiotherapy‐temozolomide chemoradiotherapy significantly prolonged the mPFS of 8.1 months in the CIK group compared with 5.8 months in the control group. Grade 3 or higher adverse events, health‐related quality of life and performance status did not differ between the two groups (104).

Immunotherapy with anti-PD-1 antibody is revolutionizing the treatment of many cancers; however, many patients do not have tumor responses to PD-1/PD-L1 inhibitors in part due to inadequate intra-tumoral T cell infiltration. CIK cells can selectively infiltrate the tumor tissue and secrete INF-γ (47), thus inducing a local inflammatory microenvironment and ultimately anti-PD-1 therapy effectiveness (112). The efficacy of CIK cells combined with anti-PD-1 therapy was investigated in metastatic RCC (105) and NSCLC (106) patients observing an mPFS of 15 and 19.3 months, respectively.

To improve CIK cell-based therapies, preclinical studies have shown that the combination with pulsed dendritic cells (DC) leads to a significant improvement in antitumor activity when compared with CIK or DC treatment alone. Indeed, DC-CIK immunotherapy allows delivery of both DCs, which have potent capacity for antigen presentation and induction of adaptive immune responses, and CIK cells with cytotoxic activity. Moreover, the DC-CIK interaction stimulates the proliferation and antitumor activity of CIK cells through the secretion of IL-12, IFN-γ, and other cytokines (113, 114). Indeed, in a 10 year follow-up study, Lin et al. showed that DC-CIK treatment can significantly improve DFS and OS compared to the control group, in patients with stage IV breast cancer who only rely on chemotherapy regimen option (5-year DFS 42% vs 30% and 5-year mOS 44% vs 29%). Additionally, multivariate analysis confirmed that DC-CIK therapy significantly and independently reduced the risk of post-operative disease progression and patient death (107). Also, a comparable improvement in patient survival and therapeutic efficacy was highlighted in a prospective study in advanced pancreatic cancer patients (NCT01781520), where the combination of oral chemotherapy S-1 and DC-CIK immunotherapy resulted in circulating immune effectors modulation and prolonged 6-month OS and PFS (62.2% and 41.6%) when compared with the DC-CIK (18.2% and 9.09%) and chemotherapy (25% and 0%) groups (108). Interestingly, Li et al. analyzed 70 studies involving 6743 colorectal cancer (CRC) patients in a systematic review and meta-analysis. Results indicate that CIK or DC-CIK therapy improves OS, PFS, and ORR compared to standard treatment, without increasing toxicity. Overall, the study from Li et al. reported that, co-treatment with DCs did not improve clinical outcomes over CIK therapy alone. These findings underline the intrinsic therapeutic efficacy of CIK cells and suggest that in this setting the combination with DC therapy may not provide additional benefit (109).

### 4.4 CAR-CIK cell therapy

CIK cells have been also used as a promising platform for genetic modification with CAR molecules targeting CD123^+^/CD33^+^ acute myeloid leukemia (AML) (115), CD19^+^ acute lymphoblastic leukemia (ALL) (116), ErbB2^+^ rhabdomyosarcoma (RMS) (117), CSPG4^+^ (118) and CD44v6^+^ (119) soft-tissue sarcomas (STS), showing an increased cytotoxicity compared to untransduced CIK cells. CAR-modified CIK cells would exert anti-tumor activity both by intrinsic NKG2D-mediated and CAR-specific targeting (120). In 2020, Magnani et al. reported for the first time the administration of CAR-engineered CIK cells in a clinical setting (110). The multicentric phase I/II clinical trial (NCT03389035) aimed to assess the safety and feasibility of infusing allogeneic CARCIK-CD19 in patients with Acute B Lymphoblastic Leukemia (B-ALL) relapsed after HSCT. Four pediatric and nine adult patients were infused with a single dose of CAR-CIK cells. Six out of seven patients who received the highest doses achieved CR and CR with Incomplete blood Count Recovery (CRi) at day 28. Grade 1 and grade 2 CRS toxicities were reported only at the highest CARCIK-CD19 dose. Impressively, no GvHD, neurotoxicity, or dose-limiting toxicities were observed (110). This study demonstrated that even when modified with CAR molecules, CIK cells show a better safety profile when compared to CAR-T lymphocytes.

## 5 Innovative combination strategies

**Figure 2:**
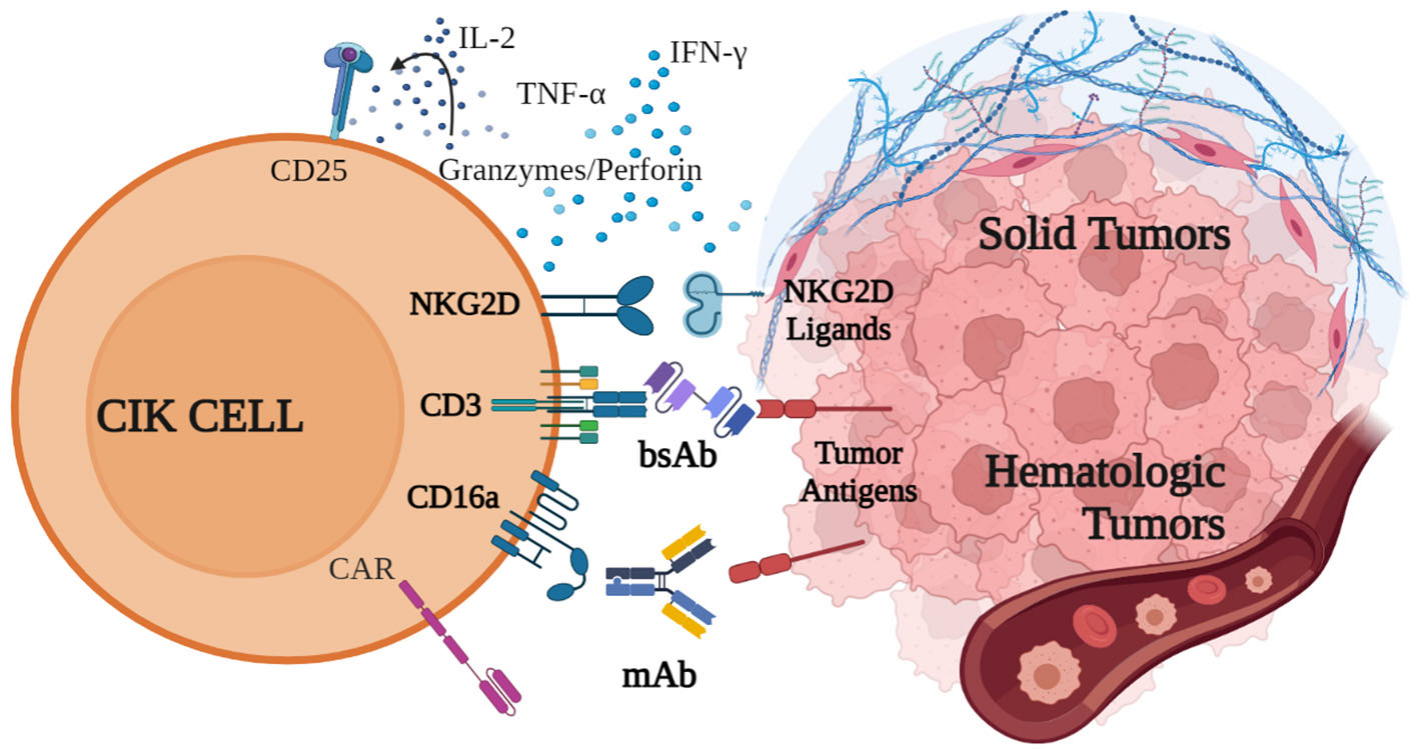
Innovative combination strategies with CIK cells. CIK cell cytotoxic activity against both hematological and solid malignancies is mainly mediated by the binding of NKG2D, which recognizes specific receptors on tumor cells, and by the release of cytokines, such as IFN-γ, IL-2 and TNF-α, perforin and granzymes. CD16a and CD3 can be engaged by monoclonal and bispecific antibodies, which bind to tumor antigens and trigger CIK cell cytotoxicity. Additionally, CIK cells can be engineered to express CAR receptors. Created with BioRender.com.

CIK cells have demonstrated all their potential in the wealth of pre-clinical and clinical studies reported in this review. This cell population offers even more possibilities to develop innovative therapeutic approaches when used in combination strategies. Besides the combination with chemotherapy, anti-PD-1 antibodies, and DCs, which have been already demonstrated to be effective in many clinical trials, the simultaneous application of CIK cell therapy and mAbs or bispecific antibodies (bsAb) (121–123) allows to amplify the targeting as well as the efficacy of the treatment (**Figure 2**) (124). Especially in solid tumors, which are highly heterogeneous and contain transformed tumor cells supported by stromal cells, combinatorial approaches may be promising to improve trafficking, persistence, proliferation and cytolytic activity of the immune infiltrate in the tumor site (125, 126).

CIK cells can be used in combination with mAbs to redirect their cytotoxic activity in an antigen-specific manner. As previously described in this review, the binding of CD16a expressed on CIK cells is able to exert a potent ADCC (27). In the first report describing this strategy, Cappuzzello et al. showed in preclinical studies that CIK cells engaged with anti-EGFR (Cetuximab) or anti-Her2 (Trastuzumab) mAbs exerted a potent ADCC against ovarian and breast cancer cell lines, leading to an increased lytic activity and a greater therapeutic efficacy *in vivo* (27). The combination of CIK cells and mAbs was also demonstrated to be effective in hematological malignancies. In preclinical studies, CIK cells could be efficiently retargeted against B-cell cancer lines and autologous tumors when combined with anti-CD20 mAbs Rituximab or Obinutuzumab (OBI), demonstrating high cytotoxicity (33). Remarkably, the CIK+OBI combined strategy has been recently evaluated for the first time in a patient with a diffuse large B-cell lymphoma (DLBCL) relapsing after four lines of therapy, including CAR-T tisa-cel. The patient did not show any infusion reaction, signs of cytokine release syndrome, or neurotoxicity. The only treatment-related adverse event was a transient reduction of platelet count after OBI. The efficacy evaluation with PET scan showed a decrease in the number of involved sites with a unique stable residual node, indicating a partial response to the progressive disease (127).

Along with CD16a, CIK cells also express CD3 and CD5 (128), which can be triggered by bsAbs (**Figure 2**). The retargeting with the CD5 on CIK cells was demonstrated with the bsAb CD5xCD19 (HD37xT5.16) (128), and CD5xCD20 bsAb (BL-01) (129). In both combinations, redirected CIK cells showed an increased cytotoxicity against CD19^+^ and CD20^+^ target tumor cells, respectively. Importantly, the same group demonstrated that CIK cells expanded from cord blood or PBMCs and then combined with Blinatumomab (CD3xCD19), significantly increased their killing capacity against an aggressive Ph^+^ CD19^+^ acute lymphoblastic leukemia PDX model in NOD-SCID mice, without signs of toxicity or GvHD (130).

The use of ACT with CIK cell alone was already demonstrated to be effective in gastric cancer (131), but the use in combination with CD3xEGRF bsAb improved their cytotoxicity both *in vitro* and *in vivo* mouse models (132, 133). This approach was confirmed against other tumor cell lines expressing EGFR, such as colon, lung, colorectal, cervical and prostate cancer, and also against glioblastoma (134). Regarding ovarian cancer, due to the high expression of Her2 on tumor cells, CIK cells were also combined with the bsAb CD3xHer2 both *in vitro* and *in vivo* (135, 136).

Remarkably, a promising Phase II clinical trial demonstrated the clinical efficacy and safety of the combination of CIK cells with anti-CD3-MUC1/CEA/EpCAM/GPC3 bsAbs in primary hepatocellular carcinoma (NTC 03146637) (137). Indeed, no significant changes in the biochemical indicators and no grade 3 adverse reactions were observed; moreover, the concentration of tumor markers was significantly decreased (137).

Nowadays, immune checkpoints (IC) inhibition has become an important strategy for cancer immunotherapy, since it aims at blocking the immune tolerance mechanisms and restoring T cells antitumor function (138). Among the most critical IC molecules, TIM-3, LAG-3, CD200R and BTLA are strongly expressed by CIK cells, compared to PD-1 or CTLA-4 which are rarely expressed (130, 139). However, their role in CIK cell context is still controversial due to the both inhibitory and stimulatory effects described in cytotoxicity (138–140). The high expression level of some IC with respect to others could be related to unclear biological functions, which need to be better elucidated making at the moment challenging a therapeutic application in combination with CIK (138–140).

The approach combining CIK cells with mAbs or bsAb can find wide implementation and application using other therapeutic compounds already approved or in the late stage of clinical development. Importantly, these approaches could be immediately translated to the clinical setting in an extremely vast array of different tumors simply by changing the desired mAb or bsAbs, with no need to genetically modify the cells.

## 6 Conclusions and final considerations

In the ACT landscape, CAR-T cell limitations open opportunities for improvement and for the potential development of additional therapeutic options. CIK cells could represent a more than attractive alternative, as they can be manufactured using an extremely simple, time-saving and cost-effective approach that requires a minimal need for technical interventions and avoids the use of expensive devices. This procedure could be realistically realized in local cell factories, thus providing easier access to the therapy in local hospitals and moving forward from centralized manufacturing facilities.

Although developed more than 30 years ago, CIK cells are not yet a commercial product remaining confined to investigations in academia, if compared to CAR-T cells that have obtained accelerated approval from FDA and are now marketed worldwide. Noteworthy, the vast majority of clinical studies evaluating CIK cell therapy have been performed in China, and many of them are reported only in Chinese, whereas in the rest of the world their study is restricted to few isolated academic centers. In our opinion, the geographic distribution of CIK cell clinical studies is likely the main reason explaining why CIK cells have not yet obtained approval from FDA. Second, pharmaceutical companies are not involved in CIK cell development probably because of the lack of an adequate patent coverage, which limits the appeal for investments.

Moreover, the extreme heterogeneity of CIK cell clinical studies makes a thorough understanding of their real clinical efficacy more challenging. Starting from the manufacturing process, many different protocols have been reported, including the media, concentration of stimuli, and cytokines used, and timing of cytokines addition in culture (23). The antitumor ability, immune phenotypes, and cytokine secretion of the resulting CIK cells may be slightly different. Furthermore, characterization of the cell therapy product prior to infusion is generally not provided in the clinical trial reports, and the combination of CIK cells with other therapies further complicate the data interpretation. Undoubtedly, a higher standardization in terms of study design, number of infused cells, and clinical evaluation among CIK cell clinical trials, would allow a more comprehensive comparison and understanding of the results and a better interpretation of therapeutic efficacy, thus supporting a broader application of CIK cell therapy. Overall, existing data indicate that CIK cell therapy is suitable to prevent recurrence, improves quality of life and prolongs OS as well as PFS. However, only large randomized multi-center phase III studies that rely on clinical centers in different countries, standardized procedures and uniform clinical response assessment, will have the chance to definitely establish the therapeutic potential of CIK cell approach.

## Author contributions

Each author was assigned to write a section. RS and EC planned the work and critically revised the article. All authors contributed to the article and approved the submitted version.
